# Detection and Stability of SARS-CoV-2 Fragments in Wastewater: Impact of Storage Temperature

**DOI:** 10.3390/pathogens10091215

**Published:** 2021-09-18

**Authors:** Rudolf Markt, Markus Mayr, Evelyn Peer, Andreas O. Wagner, Nina Lackner, Heribert Insam

**Affiliations:** Department of Microbiology, University of Innsbruck, 6020 Innsbruck, Austria; markus.mayr@uibk.ac.at (M.M.); Evelyn.Peer@i-med.ac.at (E.P.); Andreas.Wagner@uibk.ac.at (A.O.W.); nina.lackner@i-med.ac.at (N.L.); Heribert.Insam@uibk.ac.at (H.I.)

**Keywords:** SARS-CoV-2, storage, wastewater, stability, freezing

## Abstract

SARS-CoV-2 wastewater epidemiology suffers from uncertainties concerning sample storage. We show the effect of the storage of wastewater on the detectable SARS-CoV-2 load. Storage at 4 °C for up to 9 days had no significant effect, while storage at −20 °C led to a significant reduction in gene copy numbers.

## 1. Introduction, Aims and Methods 

In the context of the global COVID-19 pandemic, the quantification of severe acute respiratory syndrome coronavirus 2 (SARS-CoV-2) fragments in wastewater offers the opportunity to monitor the level of infection in large populations, independent of apparent symptoms [1,2]). With the growing number of SARS-CoV-2 wastewater studies, we need comprehensive knowledge on common storage procedures for raw wastewater to generate valid data from sewage surveillance. Temperature, as a central environmental parameter, is a main driver of microbial decay and significantly alters the persistence of viruses in wastewater. Thereby, storage at lower temperatures (<4 °C) increases the persistence of coronaviruses in wastewater [3]. The aim of this investigation was to compare the effect of the most common storage temperatures (≤4 °C) of wastewater samples, +4 °C and −20 °C [4,5], on the detectability of SARS-CoV-2 gene copy numbers.

Therefore, we analyzed 24 h composite samples of raw influent wastewater from the wastewater treatment plant (WWTP) Zirl, Tyrol, Austria (19 April 2020, 30,000 population equivalents (PE)) and the WWTP Siggerwiesen, Salzburg, Austria (4 October 2020, 680,000 PE). The wastewater from the WWTP Zirl was roughly composed of 1/3 industrial and 2/3 domestic wastewater, with a catchment length (main collector) of 50 km. The sample from this plant was taken during dry weather with a mean ambient temperature of 15.2 °C. Meanwhile, the wastewater from the WWTP in Salzburg was mainly composed of domestic wastewater, with a catchment length (main collector) of 140 km. Sampling for this site was conducted during dry weather with a mean ambient temperature of 11.4 °C. The former samples (Zirl) were pasteurized prior to analysis due to uncertainties of the safety status of the wastewater at this time, while the latter (Salzburg) remained unpasteurized. Pasteurization of the wastewater involved an exposure of the samples to 60 °C for 1.5 h prior to sample processing [6]. The investigated storage conditions are summarized in Table 1.

For SARS-CoV-2 RNA extraction, we modified the protocol from Wu et al. [2]. In a first step, larger particles were removed to decrease the amount of non-viral RNA and PCR inhibitors. For this purpose, 40–70 mL of wastewater was transferred to centrifugation tubes and centrifuged at 4500× *g* for 30 min at 4 °C. To precipitate viral fragments, the resulting supernatant was immediately transferred into a fresh tube containing 10% *w*/*v* polyethylene glycol (PEG) 8000 (CarlRoth, Karlsruhe, Germany) and 2.25% *w*/*v* NaCl. The Reax2™ overhead shaker (Heidolph, Schwabach, Germany) was used until both additives were dissolved within a few minutes. Subsequently, the samples were centrifuged at 12,000× *g* for 99 min at 4 °C to obtain a pellet containing the viral fragments. The supernatant was removed in two steps. First, most of the supernatant was carefully decanted, and then, after additional centrifugation at 12,000× *g* for 5 min, a pipette was used to remove the remaining fluid.

Following the precipitation of the viral fragments, pellets from the Zirl samples were resuspended with 800 µL TRIzol^®^ (Invitrogen, Waltham, MA, USA), and TRIzol^®^-chloroform extraction was performed. For the Salzburg samples, we substituted hazardous TRIzol^®^ with 800 µL lysis buffer (Monarch™ total RNA Miniprep Kit, NewEnglandBiolabs, Ipswich, MA, USA). The aqueous, pale phase from TRIzol^®^-chloroform extraction or the pellet resuspended in lysis buffer was purified according to the manufacturer protocol of the Monarch™ total RNA Miniprep Kit with non-enzymatic gDNA removal. RNA was eluted in 40 µL RNase-free water.

RNA concentrations of the templates were quantified via a Nanodrop, and extracts with RNA concentrations above 200 ng µL^−1^ were diluted as needed. RNA copy numbers were determined using the N1 primers/probe according to the CDC protocol [7] targeting the nucleocapsid gene of SARS-CoV-2. RT-qPCR reactions contained the following per 20 µL: 10 µL Luna Universal Probe One-Step Reaction Mix (2X) from NEB, 1 µL Luna WarmStart^®^ RT Enzyme Mix (20×) from NEB, 0.8 µL primer (final concentration 0.4 µM), 0.4 µL probe (final concentration 0.2 µM), 2 µL PCR-grade water, and 5 µL template. Analyses were conducted on a RotorGene cycler (Qiagen, Hilden, Germany). After an initial reverse transcription at 55 °C for 10 min, followed by 95 °C for 1 min of denaturation, 45 cycles of 95 °C for 10 sec and 60 °C for 40 sec were performed. To calculate copy numbers, a plasmid standard containing the N gene of SARS-CoV-2 (2019-nCoV_N_Positive Control, IDT, Leuven, Belgium) was used. All variants were processed in parallel (*n* ≥ 3) and were tested for significant differences against day 0 using the Mann–Whitney U Test (α = 0.05) in the software package Past 4.03 [8].

Within all samples stored at 4 °C, variation coefficients spanned from 2% to 51%, with a median of 37%. This variance inhomogeneity may be explained by the heterogeneity of the influent wastewater and by the accumulation of inaccuracies during the multi-step extraction protocol. Large variance and inhomogeneity were reported earlier by Wu et al. [9] and seemed to be independent of the method of viral fragment concentration as reported by Ahmed et al. [2]. Pasteurization of the former samples (Zirl) may have had an impact on the recovery and analysis of SARS-CoV-2 from wastewater. Pecson et al. [10] showed that pasteurization may lead to a slight increase in the recovery of SARS-CoV-2 fragments. Nevertheless, samples were pasteurized right before sample processing, and storage conditions were equal for all variants.

## 2. Results and Discussion

**Figure 1:**
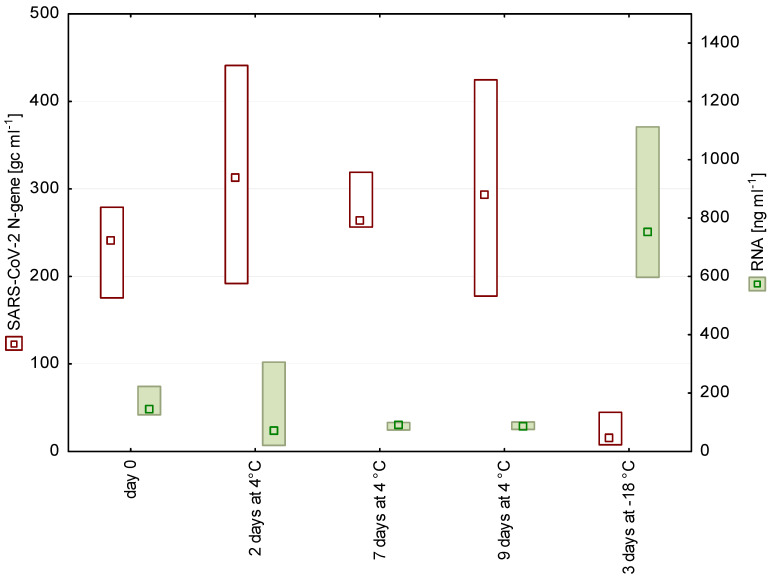
N gene copy numbers and RNA concentrations detected in wastewater from a WWTP in Salzburg after 0, 2, 7, and 9 days of storage at 4 °C as well as after freezing (*n* = 4, median, box: min-max).

**Figure 2:**
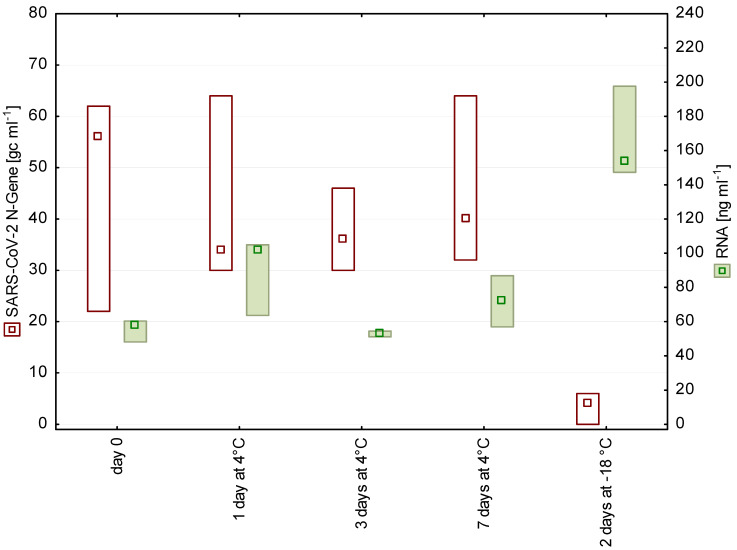
N gene copy numbers and RNA concentrations detected in wastewater from a WWTP in Tyrol after 0, 1, 3, and 7 days of storage at 4 °C as well as after freezing (*n* = 3, median, box: min-max).

Short-term storage of wastewater for up to 9 days at 4 °C had no significant effect on the number of detectable SARS-CoV-2 fragments (Figure 1 and Figure 2). These findings are in accordance with earlier studies on enveloped viruses [11,12]. In contrast to our storage experiments, Ahmed et al. [3] chose a spike-in approach, using high loads of gamma-irradiated SARS-CoV-2 virions (approximately 6.7 × 10^5^ gc mL^−1^), and stated a decay rate of approximately 8% per day at 4 °C.

Freezing–thawing of samples led to a significant loss of the signal. A possible reason is that the freeze–thaw cycle disrupts cells, which is also reflected in the increased RNA concentration in the frozen samples (Figure 1 and Figure 2). The release of cell contents possibly includes also proteases and RNases, which may impair the subsequent SARS-CoV-2 detection.

In conclusion, we recommend storing wastewater samples for SARS-CoV-2 analysis at 4 °C upon analysis and not freezing them.

## Figures and Tables

**Table 1:** Experimental design.

WWTP	Sampling Date	Pasteurization	Storage at −18 °C	Storage at 4 °C
Zirl, Tyrol	19 April 2020	yes	2 days	0, 1, 3, 7 days
Siggerwiesen, Salzburg	4 October 2020	no	3 days	0, 2, 7, 9 days
